# Incidence and burden of anal cancer—time to fight the growing disparities

**DOI:** 10.1016/j.esmogo.2025.100147

**Published:** 2025-02-24

**Authors:** E.S.L. Pedersen, D. Verschoor, E. Segelov

**Affiliations:** 1 Department of Clinical Research, University of Bern, Bern, Switzerland; 2 Department of Radiation Oncology, Inselspital, Bern University Hospital, University of Bern, Bern, Switzerland

**Keywords:** anal cancer, anal squamous-cell carcinoma, epidemiology, incidence, global disparities

## Abstract

Anal cancer (AC) is rare, but its incidence is increasing, although at different rates across countries and population groups. The increase in AC incidence is primarily due to the increased prevalence of human papillomavirus (HPV), which causes 90% of squamous-cell carcinoma (SCC), the most common AC histology subtype. Understanding estimates of incidence across countries and risk groups is essential for estimating future disease burden and predicting cancer care resource allocation. In this review, we summarize the latest literature with an emphasis on global disparities and challenges in AC prevention. Globally in 2020, >30 000 people were diagnosed with anal SCC, with two-thirds of cases occurring in North America and Europe. AC incidence varies greatly between population groups; higher incidence is associated with older age, females, anal sexual intercourse, immunosuppression [particularly people living with human immunodeficiency virus (HIV) and solid organ transplant recipients], and prior HPV-related gynecologic (pre-)cancers. Primary prevention of AC relies on preventing HPV infection by vaccination and secondary prevention relies on screening and treatment of high-grade squamous intraepithelial lesions (HSILs). Globally, there are considerable disparities in AC. While its incidence is currently highest in high-income countries, it is expected to increase in low-income countries (LICs) in the future, due to higher HPV prevalence and lower HPV vaccine coverage. Continued effort is essential to gather data on prevalence and incidence, especially in LICs, to strengthen vaccination programs globally and to promote research networks around the world for better evidence on screening and treatment.

## Highlights

• AC is rare, yet its incidence is increasing in most countries.
• HPV is the main cause of rising AC incidence.
• Risk factors include age, female sex, anal intercourse, and immunosuppression.
• Incidence data come mainly from high-income countries; we need better global data.
• Strengthening HPV vaccination programs globally is essential with inclusion of males.

## Introduction

Anal cancer (AC) is a rare malignancy occurring in the mucosa-lined anal canal.^1^ The most common histology is squamous-cell carcinoma (SCC) comprising 75%-95% of cases also including low keratinized cloacogenic cancer as it is treated similarly. Adenocarcinoma comprises around 5%-15% of cases, although some authors consider these all as arising from the rectum and their prognosis and treatment should be as per histology not site.^2^ Very rare histologies, including melanoma, lymphoma, neuroendocrine, gastrointestinal stromal tumors, and even rarer metastases from other sites, constitute <5%.^3^, ^4^, ^5^, ^6^, ^7^, ^8^ AC usually presents as local or locoregional disease with a high 5-year overall survival >85%^9^; metastatic AC is rare, both *de novo* and as relapsed disease, and is associated with much lower survival than local disease with reported 5-year survival of 61% for stage 3 and 18% for stage 4 disease.^7^^,^^10^ As with cervical cancer, AC is of viral etiology with the pathogen human papillomavirus (HPV) strongly associated (up to 90% of cases), in particular HPV16.^11^ Data from GLOBOCAN, which reports cancer statistics from 185 countries, recorded 690 000 new cancer cases worldwide in 2018 attributable to HPV, of which 29 000 were anal SCC.^12^

Understanding estimates of incidence across countries and risk groups is essential for estimating future disease burden and predicting cancer care resource allocation. In this review, we summarize the latest literature with an emphasis on global disparities and challenges in AC prevention, focusing on mucosal AC.

## Epidemiology

AC incidence is increasing, with the rise attributed to SCC histology. The increase is primarily due to the increased prevalence of HPV, which causes 90% of SCC, but is also affected by increased absolute numbers of men who have sex with men (MSM) living with human immunodeficiency virus (HIV) and organ transplant recipients.^13^ Anal adenocarcinoma incidence is stable or declining.^6^^,^^14^ Many studies report incidence rates (IRs) only for SCC, while others report on all histology types. In the following, we distinguish between anal SCC and AC incidence estimates based on what was reported.

Globally, an estimated 30 416 people were diagnosed with anal SCC in 2020.^15^ In the United States alone, the Surveillance, Epidemiology, and End Results (SEER) program reported 9760 new cases in 2023, corresponding to an age-adjusted IR of 1.9 per 100 000 person-years, and comprising 0.5% of new cancer diagnoses.^16^ The incidence rose from 1.20 per 100 000 person-years in 2001-2005 to 1.56 per 100 000 person-years in 2011-2015, with highest increases in women and people aged ≥50 years.^17^ Data from the Cancer Incidence in Five Continents (CI5) database showed differences between countries, with highest overall IRs in Denmark, France, Germany, United States, Switzerland, UK, and Australia and lowest rates in Japan, Turkey, China, Philippines, and Jordan.^14^^,^^15^^,^^18^ The steepest 5-year increases in age-standardized IR between 1988 and 2012 were observed in the Netherlands (38%), Denmark (20%), and UK (20%).^14^

### At-risk populations

AC incidence varies greatly between population groups.^19^, ^20^, ^21^, ^22^ Older age, females, anal sexual intercourse, immunosuppression (particularly people living with HIV and solid organ transplant recipients), and prior HPV-related gynecologic (pre-)cancers are associated with a higher incidence.

In most countries, the age-standardized IR is around double for women.^6^^,^^15^^,^^20^^,^^23^, ^24^, ^25^ This is almost certainly due to HPV coinfection of the cervix and anal epithelium.^11^ For example, in Denmark between 2013 and 2018, the age-standardized IR of SCC was 2.21 and 0.91 per 100 000 person-years comparing women and men, respectively.^7^ Meta-analysis data on AC from 2008 to 2012 show age-standardized IRs of 2.16 and 2.08 per 100 000 person-years for women in Germany and Switzerland, respectively, but lower rates in Japan and Spain (0.26 and 0.41, respectively).^18^ The increased incidence of AC during the past 20 years was also steeper among women, especially those aged ≥60 years.^14^

The population group with the highest AC incidence is MSM living with HIV, resulting from exposure to HPV infection from anal receptive sexual intercourse.^26^ Data from a recent systematic review and meta-analysis showed an overall AC IR among MSM living with HIV of 85 per 100 000 person-years, with large difference by age: IR of 17 per 100 000 person-years for those aged <30 years compared with 108 per 100 000 person-years for those aged ≥60 years.^19^

Few studies exist on the incidence of AC among MSM without HIV; data so far only exist on SCC. The Multicenter AIDS Cohort Study reported a crude SCC IR of 20 per 100 000 person-years [95% confidence interval (CI) 10-40], with estimates relying on only eight cases.^27^ Similar estimates for SCC incidence were found in a recent analysis of pooled data from multiple USA databases.^28^

People living with HIV have a 10-15 times higher risk of AC than the general population. Data from a recent meta-analysis from the United States, France, the Netherlands, and Switzerland showed a pooled IR estimate of 32 (95% CI 30-35) per 100 000 among males who are not MSM and 22 (95% CI 19-24) per 100 000 among women.^19^ Data from the Australian National HIV Registry on SCC showed a crude IR of 36 (95% CI 30-41) per 100 000 person-years between 1982 and 2012, with increasing trend over time and higher risk in older age.^29^ Past infection with hepatitis B, low CD4+ cell counts, older age, and time since HIV or acquired immunodeficiency syndrome diagnosis are associated with a higher cumulative incidence of AC.^27^^,^^29^^,^^30^

Few analyses exist reporting risk of AC among solid organ transplant recipients. A meta-analysis of the six existing studies showed an overall SCC IR of 7 per 100 000 person-years.^21^ Data from the United States Transplant Cancer Match Study show higher incidence with time since transplantation, older age, and female sex.^19^

High-grade squamous intraepithelial lesion (HSIL) is a precursor lesion for AC. Reported progression rate 5 years after HSIL to AC ranges between 4% based on data from Denmark and 9.5% based on SEER data.^31^^,^^32^ Autoimmune disease, genital warts, and HIV are risk factors for progression to AC.^33^ Similarly, individuals with prior HPV-related cancer are at higher risk of AC and are therefore good candidates for focused screening studies.^19^^,^^34^

## Preventing AC

Primary prevention measures, in particular HPV vaccination, are expected to prevent most anal SCC in the future. Due to the long time lag and the rarity of anal SCC, no data are currently available for the general population for AC. However, first data show that HPV vaccine can prevent HSIL. In an ecological study from Denmark, Baandrup et al. used whole-population registries to include all Danish women aged 17-32 years between 2006 and 2021 to study if HPV vaccine prevented occurrence of HSIL.^35^ Among 968 881 women contributing 7.2 million person-years, 37 cases of HSIL occurred among the unvaccinated, compared with <5 among those fully vaccinated before 17 years of age, corresponding to a hazard ratio of 0.30 (95% CI 0.10-0.89) for vaccination. Despite the low event number, this study provides population-level evidence of the promising effect of HPV vaccination. However, current evidence is conflicting about whether HPV vaccine administered after the onset of sexual activity can prevent HSIL in high-risk populations such as MSM.^36^^,^^37^

Secondary preventive measures include screening for and treatment of HSIL.^38^ Screening methods include anal cytology, analogous to survival screening methods, which utilize liquid-based cytology and HPV biomarker tests, but sensitivity differs between people living with HIV (84%-94%) and without HIV (57%-91%).^39^ High-resolution anoscopy has higher sensitivity but is costly and requires considerable expertise and time to carry out.^40^ To our knowledge, no randomized controlled trials exist evaluating the effect of screening high-risk populations for HSIL on AC risk. Cohort studies have shown reduced AC incidence among screened individuals,^41^^,^^42^ but inherently the risk of selection bias and confounding is high. One study exists on the effect of HSIL treatment on preventing progression to cancer. This United States study, published in 2022, randomized people living with HIV with HSIL to HSIL treatment (*n* = 2237) or active monitoring (*n* = 2222).^43^ During a median follow-up of 26 months, 9 AC diagnoses were observed in the treatment group (IR 173 per 100 000 person-years) and 21 AC diagnoses in the active monitoring group (402 per 100 000 person-years). The rate of progression to AC was thus lower in the treatment group compared with the active monitoring group, providing important evidence to support treatment of HSIL. A similar effect is expected in people without HIV, but future studies should confirm this. Guidelines exist recommending screening people living with HIV, MSM without HIV, and solid organ transplant recipients, but further knowledge is needed to tailor programs by age and risk categories.^44^^,^^45^

## Disparities

To date, most disparity research has focused on MSM and people living with HIV, but studies are keenly needed to address the stark gender disparity in this disease. Barriers to understanding disparity include inadequate data on a global scale and inequities in HPV vaccination.

### The problem of data availability

Due to its rarity, estimation of AC incidence requires large representative datasets, ideally covering extensive periods to analyze changes over time. Gathering such datasets is challenging in low-resource settings; hence, most evidence on incidence originates from high-income countries (HICs) that have established national cancer registries, including well-ascertained outcome data.^29^ Some studies exist from middle-to-high-income countries (HMICs) such as Thailand,^46^ but few studies originate from low-income countries (LICs),^47^ where most incidence estimates rely on few events, resulting in estimates with high uncertainty. There is a need to invest financial and human resources in establishing and sustaining cancer registration systems to report on all cancers, including rare malignancies, in low-and-middle-income countries (LMICs). This is undoubtedly a challenging task due to factors including economics, health systems, and cultural beliefs, among many.^48^

### Inequities in the fight against human papillomavirus

Despite the lack of robust data, it is likely that AC incidence is increasing more rapidly in LICs. There are several reasons for this. Firstly, the prevalence of HPV is high in many LICs, with recent estimates of 22% for men in LICs and 26% in LMICs.^49^ The high prevalence of HPV in women and limited screening programs have resulted in high incidence of cervical cancer, which is more prevalent and develops earlier than AC. Recent data showed an age-standardized IR of 9.1 per 100 000 for cervical cancer in HICs, 16.6 in HMICs, and 27.2 in LICs.^50^

Secondly, while HPV vaccination programs have been introduced in 107 countries by 2020, 74 of these still only vaccinate girls.^51^ In addition, even when offered, vaccination uptake is lower in boys in many countries, especially LMICs. One study of 17 countries reports an uptake rate of 45% among girls and 8% among boys.^52^ Full HPV vaccination coverage in LICs was estimated to be only 23% in 2019, compared with 40% in HICs. Costs remain a barrier in many countries. A study from 24 countries between 2008 and 2020 showed lower vaccine coverage in countries with government-funded vaccination programs than those funded by international non-government organizations.^52^ However, the sustainability of the latter is a future concern.

Further barriers to higher vaccine uptake include lack of awareness. A study among adults in the United States showed that only 16% of 16 000 respondents knew of the causal relationship between HPV and cancer.^53^ A better understanding of the risk of HPV infection may improve vaccine uptake.

In summary, the incidence of AC is predicted to rise based on current data on HPV prevalence, and sustainable strategies to increase HPV vaccine coverage worldwide, but especially in LMICs, are needed.

## Conclusions

AC incidence has risen in the past few decades, and current evidence suggests that incidence will continue to rise, with highest increases predicted in women and in LMICs.^50^^,^^54^ Continued efforts to gather data on the prevalence and incidence of AC are essential to better understand the burden of disease and to prioritize health care. Strengthening of HPV vaccination programs globally with the inclusion of boys is essential. Randomized controlled trials in populations at high risk of AC (e.g. MSM and people living with HIV) are needed to optimize effective screening for HSIL. Clinical research networks can help strengthen and focus research on a global scale. Only through collaboration and unified action will it be possible to reduce the increasing disparity gap in AC burden globally.
